# A case report of Lhermitte–Duclos disease revealed by psychiatric disturbances

**DOI:** 10.1186/s12991-017-0147-1

**Published:** 2017-05-30

**Authors:** Yassine Otheman, Rachid Aalouane, Chadia Aarab, Ismail Rammouz

**Affiliations:** 1 Faculty of Medicine and Pharmacy, Sidi Mohamed Ben Abdellah University, Fez, Morocco; 2 Psychiatry Department, Faculty of Medicine and Pharmacy, Sidi Mohamed Ben Abdellah University, Fez, Morocco

**Keywords:** Lhermitte–Duclos disease, Cerebellar lesion, Psychiatric symptoms

## Abstract

**Background:**

Lhermitte–Duclos disease (LDD) is a rare cerebellar lesion characterized by a hamartomatous lesion in the posterior fossa. Mainly diagnosed by MRI, the clinical presentation is usually made of neurological symptoms.

**Case presentation:**

We present here a rare case of a woman who developed depressive symptoms that inaugurated the clinical presentation of LDD.

**Conclusion:**

Psychiatric symptoms may occur in all brain lesions, delaying the diagnosis and causing therapeutic escalation. More attention should be given by practitioners to psychiatric aspects of LDD.

## Background

Lhermitte–Duclos disease (LDD) is a rare cerebellar lesion, described in 1920 by two French physicians: Lhermitte and Duclos [1]. This entity, also called dysplastic cerebellar gangliocytoma, is characterized by a hamartomatous lesion in the posterior fossa. The clinical presentation of this disease is not specific, it is usually related to intracranial pressure, cerebellar dysfunction, and cranial nerve deficits, and it is mainly diagnosed by cerebral magnetic resonance imaging (MRI) [2–4]. We present here a rare case of a woman who developed psychiatric symptoms that led to LDD diagnosis.

## Case presentation

**Fig. 1:**
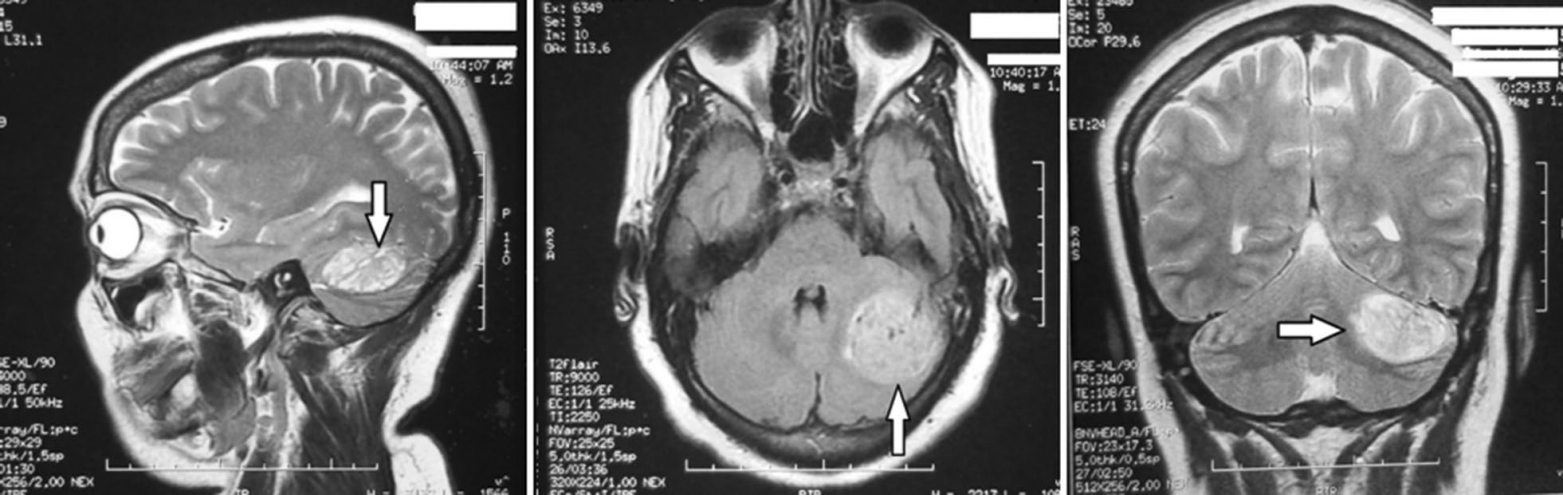
Three MRI images showing an expansive intra-axial mass of the *left* cerebellar lobe, with no mass effect on the ventricular system, measuring 37 × 35 × 23 mm

A 51-year-old lady, without psychiatric history, was referred for depressive symptoms lasting for 4 months: sadness, irritability, loss of pleasure, feeling hopeless, and insomnia. In addition to these psychic symptoms, other usual physical symptoms of depression were observed (pain and aches, lack of energy, decreased appetite, etc.). After a routine blood test, and an extended psychiatric examination, the diagnosis of a major depressive episode was made, and the patient was put on antidepressant (paroxetine 20 mg/day). One month later, the improvement was relative and several symptoms persisted, the patient started to complain of more headaches, and visual disturbances have emerged. An ophthalmic examination revealed a mild bilateral papilledema, and the neurologic examination found a mild coordination disorder (dysdiadochokinesia on the left), but no associated nausea, vomiting, or gait disturbance and a negative Rhomberg’s sign. MRI revealed an expansive intra-axial mass of the left cerebellar lobe, with no mass effect on the ventricular system and without enhancement after intravenous injection, which confirmed the diagnosis of LDD (Fig. 1).

No Cowden syndrome or familial history of the disease was found in this patient. Because of the risks of the surgery, the neurosurgeon decided to have a conservative strategy and prescribed only symptomatic treatment (methylprednisolone 64 mg/day). Two months later, the patient improved slightly, and returned to her normal life, with occasional minimal headaches and a better improvement of psychiatric symptoms with the same antidepressant treatment.

## Discussion and conclusion

Lhermitte–Duclos disease is a rare disease, with no race or sex preferences [5]. It affect usually people between 30 and 50 years old, even if it may occur in other ages [5, 6]. The prevalence of the disease is unknown: there are at least 230 cases that have been reported in medical literature, but no psychiatric symptoms were described before. Lhermitte and Duclos described for the first time the histopathology of the lesion, characterized by a loss of granular cells, purkinje cells, and white matter, and an overgrowth of cerebellar ganglion cells, which cause the thickening of the cerebellar folia [6, 7].

Lhermitte–Duclos disease may be part of Cowden syndrome, also called hamartoma-neoplasia syndrome, which associate multiple hamartomas and a high risk of malignant tumors [8, 9]; therefore, Cowden syndrome must be sought whenever LDD is diagnosed. In this patient, LDD is not associated with Cowden syndrome, and the evolution in this case is usually very progressive, without known risk of malignancy [10].

Clinically, LDD patients may remain asymptomatic or present some neurological symptoms: ataxia, headache, cranial nerve palsies, etc., while intracranial pressure and hydrocephalus may be observed in severe cases [2, 3]. However, no psychiatric symptoms were described with LDD until now.

In this patient, depressive symptoms preceded the neurological disorders. The absence of mood disorders or other psychiatric history in this patient and the late onset of depression at this age are elements in favor of the somatic origin of these psychiatric symptoms. Another important proof of this origin is the non-improvement of depressive symptoms under antidepressant alone, while adding somatic treatment (corticotherapy) led to better response to the same antidepressant medication. It is usually difficult to suspect a brain tumor when depression is the only clinical expression, and the patient have a normal neurological examination, but there are some indicators for an organic origin of such psychiatric symptoms, particularly the late onset of depression and treatment resistance [11].

Even if there are no anterior reported cases of psychiatric disturbances in LDD, and the lack of data about psychiatric manifestations in relation to cerebellar tumors, such symptoms may occur in all brain lesions, and may be underdiagnosed because of the predominance of neurological symptoms. But when psychiatric disturbances inaugurate the clinical presentation of a rare disease like LDD, they may delay the diagnosis and cause psychiatric therapeutic escalation.

The psychiatric symptoms in LDD may be explained by structural changes of the brain in this region, which could affect the whole neurotransmitter function. An absence of central benzodiazepine receptors in the lesion was proved [12], suggesting that other receptors may be affected.

In this patient, MRI was sufficient to make the diagnosis of LDD, because of the presence of typical images. Usually MRI with diffusion imaging and spectroscopy are used to confirm the diagnosis by showing the unilateral expansile lesion, with hypointense on T1 and hyperintense on T2 weighted sequences. No cystic or necrotic areas are observed, but calcifications may be present [10, 13, 14]. CT may not help since the petrous temporal bone can cause artifacts and hide details in the posterior fossa [15]. Other authors suggested that histopathologic confirmation is required to avoid misdiagnosis [4].

The decision of not going to surgery here was dictated by the fact that the patient improved under symptomatic treatment, and by the relatively high risk of damages or recurrence after surgical excision [4]. The radical surgical removal of the lesion is usually reserved for cases with severe neurological symptoms, and for young people with a well-circumscribed lesion which allow a total and safe surgery [16, 17]. However in symptomatic elderly patients, partial resection may be recommended to reduce mass effect and avoid surgical complication [18].

This case shows the importance of imagery, especially MRI, in the diagnosis of neuro-organic disease that may occur with psychiatric appearance. More attention should be given by practitioners to psychiatric aspects of LDD.

## Abbreviations

CT: computerized tomography
LDD: Lhermitte–Duclos disease
MRI: magnetic resonance imaging
